# Interoperable and accessible census and survey data from IPUMS

**DOI:** 10.1038/sdata.2018.7

**Published:** 2018-02-27

**Authors:** Tracy A. Kugler, Catherine A. Fitch

**Affiliations:** 1 University of Minnesota, IPUMS Center for Data Innovation, 225 19th Ave. S., 50 Willey Hall, Minneapolis, Minneapolis 55455, USA

**Keywords:** Social sciences, Health care, Geography

## Abstract

The first version of the Integrated Public Use Microdata Series (IPUMS) was released to users in 1993, and since that time IPUMS has come to stand for interoperable and accessible census and survey data. Initially created to harmonize U.S. census microdata over time, IPUMS now includes microdata from the U.S. and international censuses and from surveys on health, employment, and other topics. IPUMS also provides geo-spatial data, aggregate population data, and environmental data. IPUMS supports ten data products, each disseminating an integrated data collection with a set of tools that make complex data easy to find, access, and use. Key features are record-level integration to create interoperable datasets, user-friendly interfaces, and comprehensive metadata and documentation. The IPUMS philosophy aligns closely with the FAIR principles of findability, accessibility, interoperability, and re-usability. IPUMS data have catalyzed knowledge generation across a wide range of social science and other disciplines, as evidenced by the large volume of publications and other products created by the vast IPUMS user community.

## Introduction

IPUMS provides census and survey data from around the world integrated across time and space. IPUMS integration and documentation makes it easy for researchers to study change, conduct comparative research, merge information across data types, and analyze individuals within family and community context. The data and services are available through online data access systems free of charge for registered users. IPUMS began in the early 1990s to enable the use of U.S. census microdata for temporal research and has since expanded to encompass global individual-level and aggregate census data stretching as far back as the eighteenth century; survey data on topics including health, time use, education, and work; and environmental data.

Microdata are at the heart of IPUMS. Microdata are the individual responses to a census or survey, with each record containing responses to all questions for one particular person. A key feature is that these individual records are organized into households, capturing relationships among household members and often additional information about the housing unit. These data are enormously flexible and powerful, but are often not readily compatible across censuses from different times and places due to differences in record structure, questions asked, and possible responses.

In the early 1990s, social scientists at the University of Minnesota created consistently formatted versions of portions of the U.S. census microdata for six U.S. decennial years. The first version of IPUMS (which originally stood for Integrated Public Use Microdata Series), addressed several key challenges in working with microdata from multiple censuses^1,2^. The record structure was designed to be consistent over time by including every variable that occurred in any of the censuses. Numeric coding systems were reconciled so that the same response was always coded with the same value (e.g., for the sex variable, male was always coded as 1). Composite coding schemes were implemented to handle variables for which the set of possible responses changed over time (e.g., race). Composite codes provide lowest common denominator codes for general categories that can be identified in all years and detailed codes for categories as they existed in individual years. Extensive documentation was prepared to provide users with usage guidelines, descriptions of variables, information on comparability across years, background on census procedures, and technical information^3^.

The first version of IPUMS was distributed in 1993 via FTP as ASCII files, but a web-based data access system was developed shortly thereafter and launched in 1996^4^. The data access system allowed researchers to select the census years, variables, and sub-populations needed for their particular research question. Throughout the system, hyperlinks provided access to the specific pieces of documentation relevant to particular data elements. Users were able to download customized data files along with syntax files for use in major statistical packages.

The techniques, tools, and software developed for U.S. census microdata are also applicable to other types of microdata, and over the years IPUMS added several related projects. In the early 2000s, IPUMS expanded beyond U.S. population censuses with IPUMS International and the North Atlantic Population Project (NAPP)^5^. IPUMS International recognized that microdata from around the world, especially when harmonized under the IPUMS model, would be a valuable scientific resource. These data were also at great risk of loss, especially in developing countries, because they were seldom used for anything beyond compiling published tables. By securing perpetual agreements for data dissemination from National Statistical Office partners, including provisions for uniform nondisclosure rules to protect the confidentiality of respondents, IPUMS International has rescued and made available hundreds of millions of census microdata records^6^. NAPP capitalized on collaborations with genealogical organizations and other efforts to digitize historical microdata for several North Atlantic countries. Because NAPP datasets include records of every individual enumerated in the census, they provide rich opportunities for linking individuals across censuses for detailed analysis over time^7^. Expanding beyond census microdata, additional projects applied IPUMS techniques to survey data, including the U.S. Current Population Survey (IPUMS CPS), the National Health Interview Series (IPUMS Health Surveys), time use surveys from the U.S. and around the world (IPUMS Time Use), and the Demographic and Health Surveys (IPUMS DHS)^8^.

The principles of integrating data from multiple data sources, making complex data easier to access and use, and compiling comprehensive metadata and documentation also provided the foundation for projects incorporating other types of data. The National Historical Geographic Information System (NHGIS) assembled all machine-readable aggregate data tables published by the U.S. Census Bureau since 1790, along with the geographic boundary files defining the units described in the tables^9^. To facilitate analysis over time, NHGIS provides time series that track compatible tables across multiple censuses and address changes in geographic units^10^. IPUMS Terra extended the notion of data integration to work across microdata, aggregate data, and raster data, making population data from IPUMS projects interoperable with environmental data on land use, land cover, and climate^11^.

Many of these products were originally independently branded. As part of a comprehensive data preservation and organizational sustainability effort, the products are now all part of the IPUMS Center for Data Integration (www.ipums.org). This rebranding acknowledges the common principles underlying each of the products and the common infrastructure that supports them. Users can be assured of the consistent quality of data and documentation that is the IPUMS hallmark.

## Results

Although the first release of IPUMS data in 1993 predates the FAIR guiding principles, the IPUMS philosophy has always been consistent with the principles of Findability, Accessibility, Interoperability, and Re-usability^12^.

### Integrated public use microdata series goals and values

IPUMS originally stood for Integrated Public Use Microdata Series, and the components of that name represent the goals and values of the IPUMS organization. IPUMS began by integrating data using variable-level harmonization, making microdata codes consistent across time. The theme of integration has since been extended to make microdata codes consistent across countries, to harmonize aggregate data over time, and to integrate across microdata, aggregate data, and raster data. Integration efforts have always been supported by rich metadata enabling users to understand the details of the original source data, how the integration was conducted, and limitations or cautions to keep in mind. Typical IPUMS metadata include variable descriptions with comparability discussions and warnings for users, codes and labels, frequencies, universe statements and links to questionnaire text. The metadata are stored in machine-actionable databases, and are accessible both while browsing and selecting data in our web-based data access applications and in codebooks tailored to and delivered with each extract.

From the beginning, IPUMS was developed as a public good for researchers. While most of the data are available from other sources, IPUMS adds value by making the data more accessible and easier to use. IPUMS strives to make data as public as possible, while honoring requirements of data providers. (For example, IPUMS International follows more stringent registration conditions to meet the requirements of the national statistical agency partners.) Data are made available as soon as possible without embargo periods, and all data are free of charge. The IPUMS user community also plays an active role in contributing to this public good. Users frequently contact IPUMS with questions, data errors and fixes that improve the data.

Finally, the data were designed as a series: interoperable datasets to facilitate analyses of change over time. Before the development of IPUMS, the public use microdata samples of the U.S. census were a set of separate snapshots that were not interoperable. IPUMS USA allowed users for the first time to study more than 100 years of social, demographic and economic history of the United States. IPUMS now provides data interoperable not just across time, but also across places and even data types.

### IPUMS adherence to FAIR principles

IPUMS data are *findable* through a variety of mechanisms. The data are disseminated through a series of public facing web applications. Each application is accompanied by thorough documentation, which enables potential users to find the sites through web searches. Within each web application, the user interface is carefully designed to ensure that users can locate the specific data they need for their research. We are working with the Inter-university Consortium for Political and Social Research (ICPSR) to include every IPUMS product in the ICPSR catalogue with project-level metadata and links to the appropriate IPUMS website. IPUMS staff and enthusiastic users also actively promote the data resources through workshops and conference presentations, publications, use in classrooms, blogs, and social media.

In addition, all IPUMS data products are now assigned a digital object identifier (DOI). The DOIs change annually as new versions of the data are released, usually with additional datasets and overall improvements to the product. For the current version, the DOI resolves to the data access website, where users can browse metadata and download data. The DOI for previous versions resolves to an archive page describing that version, noting significant changes made between versions, and directing users to the current version of the data. Users can contact IPUMS for older versions of the data, which are archived annually when the DOI changes.

IPUMS data are *accessible* for a wide range of users. Anyone may browse the metadata made available through the IPUMS web applications. We strive to make the interfaces as easy to use as possible, enabling users to navigate our large data collections. In order to download data, users must create an IPUMS account. User management is consolidated across all IPUMS products, and user accounts track the products for which a user has registered and is authorized to use. Data are provided free of charge and with as few restrictions as possible.

IPUMS data are *interoperable* both internally and externally. Each IPUMS product integrates data from multiple datasets spanning times, places, and data structures. Users download data in widely used file formats, including comma separated value files, fixed-width ASCII files, binary data files in common statistical package formats, and shapefiles. Data extracts are bundled with metadata structured according to relevant community standards, including Data Documentation Initiative metadata for microdata and aggregate data tables and ISO-19115 metadata for spatial data. In addition, we are launching an IPUMS API initiative. Application programming interfaces will further extend the interoperability of IPUMS data. We anticipate developers using IPUMS APIs to create new ways to access and use IPUMS data. Likely examples include direct access through statistical packages, custom-built web interfaces for specific audiences, and data visualization applications.

IPUMS data are designed to be *re-usable* and have been used for a variety of research and policy applications. The extensive and diverse variety of population characteristics covered in IPUMS data makes them relevant for research spanning the social sciences and public health research and extends to other disciplines, including anthropology, psychology, and sustainability science. The rich descriptive metadata that are a hallmark of IPUMS allow these diverse users to identify data matching their needs and to use the data appropriately. Usage licenses are presented when users register for each product and as accounts are renewed annually. These agreements allow for derivative uses, provided IPUMS is cited. Redistribution and commercial usage require specific agreements, and are not allowed for some products due to limitations imposed by data providers.

IPUMS also supports findability, accessibility, and re-usability through an extensive program of user training and support. Each IPUMS product provides a series of brief video tutorials on the use of the data extract systems and other aspects of data use. We conduct in-person training and workshops at a variety of conferences and at the University of Minnesota. An active users’ forum allows users to learn from each other, asking and answering questions about the data. Finally, a highly responsive user support team answers phone calls and emails and monitors the users’ forum.

The data landscape and technical infrastructure has changed since 1993, but IPUMS’ emphasis on rich metadata, easy-to-use data access systems, and interoperable data remains unchanged. IPUMS is committed to providing data that are findable, accessible, interoperable, and re-usable.

### IPUMS products

IPUMS hosts ten products that share these principles. Each product disseminates an integrated data collection with a set of tools that make complex data easy to find, access, and use. We describe the products below in three categories: microdata covering the United States, international microdata, and geo-spatial data.

United States microdata

- *USA:* Microdata from decennial censuses from 1850 to 2010 and the American Community Survey, covering 2000 to the present. On-going collaborations with geneological organizations will incorporate complete census enumerations from 1790 to 1940, over 750 million records, into the collection (Data Citation 1).
- *CPS:* Microdata from the Current Population Survey (CPS), including the Annual Social and Economic Supplement (ASEC) since 1962 and basic monthly surveys from 1976 forward. The CPS is the leading source of data on labor force characteristics of the U.S. population and also includes supplements on topics such as education, fertility, and voting (Data Citation 2).
- *Health Surveys:* Microdata from the U.S. National Health Interview Survey (NHIS) and the Medical Expenditures Panel Study (MEPS). NHIS covers general health status, illness, functional limitations, access to and use of medical services, insurance coverage, and health behaviors for the period 1963 forward (Data Citation 3). MEPS provides data on health care expenditures by source of payment, type of care, health condition, and demographics for the period 1996 forward.
- *Higher Ed:* Data on the science and engineering workforce in the U.S. from 1993 to the present. Includes data from the National Survey of College Graduates, National Survey of Doctorate Recipients, and National Survey of Recent College Graduates, covering recipeints of science and engineering degrees and people working in STEM occupations (Data Citation 4).

International microdata

- *International:* International census microdata from the period since 1960. Includes 85 countries, 301 censuses, and over 672 million person records, provided by national statistical offices around the world (Data Citation 5).
- *DHS:* Microdata from the Demographic and Health Surveys (DHS) from 1980 to the present. Includes 101 samples from 23 African and Aisan countries, and covers topics including fertility preferences and contraception, marriage and sexuality, maternal and infant health, AIDS, female genital cutting, domestic violence, decision-making, health behaviors, education, work, media exposure, nutrition, household utilities, and wealth (Data Citation 6).
- *North Atlantic Population Project (NAPP):* International census microdata for the the eighteenth, nineteenth, and early twentieth centuries. Includes 36 censuses from eight countries, including 23 complete enumerations. For the U.S., Great Britain, and Norway, many samples are linked, enabling identification of individuals across multiple censuses (Data Citation 7).
- *Time Use:* U.S. and international time diary data for 1965 to the present. Time diary data are a structured narrative account of individual daily lives. Allows construction of time use variables combining activities with time of day, location, secondary activity, and the presence or absence of other people. Incorporates the American Time Use Survey Data Extract Builder (Data Citation 8), American Heritage Time Use Study Extract Builder (Data Citation 9), and Multinational Time Use Study Extract System (Data Citation 10).

Geo-spatial data

- *National Historical Geographic Information System (NHGIS):* Tabular U.S. Census data and accompanying GIS boundary files from 1790 to the present. Includes data from decennial censuses, American Community Survey, County Business Patterns, agricultural censuses, and religious bodies data. Time series and geographically standardized tables harmonize data over time (Data Citation 11).
- *Terra:* Integrated data on population and the environment, with global coverage from 1960 to the present. Includes census microdata and aggregate data from over 170 countries, land use/land cover data, and climate data. Uses location-based integration to transform data across microdata, area-level, and raster data structures (Data Citation 12).

## Discussion

**Figure 1:**
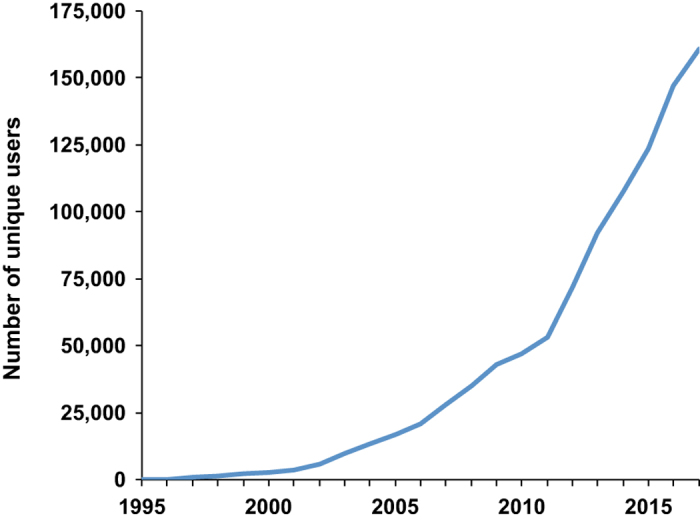
Number of registered users of IPUMS products by year. The number of users is cumulative over time. If a user is registered to use multiple IPUMS products, they are counted only once.

**Figure 2:**
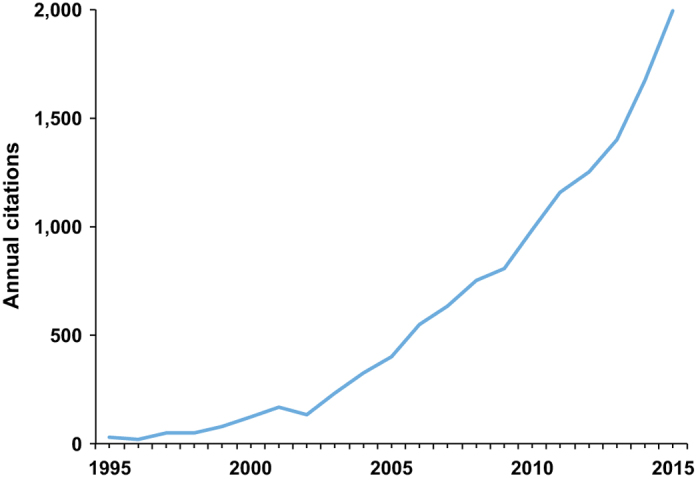
Number of Google Scholar results by year. Counts are annual, not cumulative. To capture all products, the search includes ‘IPUMS,’ ‘Integrated Public Use Microdata,’ ‘NHGIS,’ ‘Integrated Health Interview’ (former name of IPUMS NHIS), or ‘TerraPop’ (former name of IPUMS Terra).

For nearly twenty-five years, IPUMS products have been facilitating innovative research using census and survey data, accelerating knowledge generation among social scientists and allied disciplines, and preserving data at risk of loss. As of September 2017, IPUMS products have 161,000 unique users and are distributing an average of 4.3 Terrabytes of data each week (Fig. 1). Many IPUMS users are graduate students, representing the next generation of researchers. Of the more than 450 users who register each week, on average, 300 are graduate students. IPUMS users are primarily social scientists and health researchers, including economists, sociologists, demographers, epidemiologists, and geographers, but we also have users in such diverse fields as anthropology, computer science, nursing, political science, environmental science, sustainability science, psychology, and neuroscience. The research produced by these users is published in numerous articles, books, and papers, including almost 2,000 results in Google Scholar in 2015 (Fig. 2). In cooperation with users contributing citations, we maintain a searchable bibliography of publications that have used IPUMS data (https://bibliography.ipums.org). The pace of IPUMS-based publications continues to accelerate, with more than 50% of publications appearing in the past five years.

The widespread use of IPUMS clearly indicates that users find value in these data resources. The data are easy to navigate, easy to obtain, well-documented, and easy to use. In many cases, IPUMS is the only source of a dataset that has been rescued from loss, recovered from frozen microfilm or moldy data tapes, and made available to researchers^13,14^.

Part of the IPUMS mission is to preserve access to these valuable data. We are working to strengthen our data preservation program, joining DataPass and aligning our practices with community standards. We have conducted a self-assessment based on the Ten Principles for Digital Preservation Repositories^15^. We are also pursuing CoreTrustSeal Data Repository certification, based on the Data Seal of Approval and World Data Systems Core Trustworthy Data Repositories Requirements (https://www.coretrustseal.org/why-certification/requirements/). We are also undertaking a multifaceted sustainability strategy to ensure organizational and financial stability in the face of an uncertain research funding climate. IPUMS is currently funded by grants and supported by the University of Minnesota Office of the Vice President for Research. Current grants are funded by the National Science Foundation (NSF), the Eunice Kennedy Shriver Institute Child Health and Human Development (NICHD), the National Institute on Aging (NIA), and the National Institute of General Medical Sciences (NIGMS). As part of the sustainability strategy, we will pursue additional funding sources. We will also seek an organizational structure that allows key stakeholders, including data producers and data users, to contribute to IPUMS governance.

## Methods

### Consistent microdata coding

One main hallmark of IPUMS microdata products is consistent coding of variables across multiple censuses or surveys. By researching questionnaires, enumerator instructions, and other documentation, IPUMS staff identify questions in each census that address the same characteristics, such as marital status, educational attainment, or occupation. Researchers then work to map the possible responses from each census to a common coding scheme while retaining as much detail of the original response sets as possible^2,16^. Researchers create translation tables, a key piece of operational metadata, that map each original response code to a code in the standardized structure. Data processing software utilizes these translation tables to convert the source data into IPUMS data files. Some variables, such as employment status and migration status, are fairly straightforward. But other variables, notably occupation, are highly complex, requiring significant research and many complex decisions^17–19^.

### Geographic harmonization and interpolation

A major challenge in working with census data over time is that the boundaries of administrative and statistical units change from one census to the next. IPUMS addresses this challenge through two main approaches, harmonization and interpolation. Harmonized geographic units are constructed by aggregating units that change boundaries over time to create larger units with stable boundaries over a specified time period. For example, if a unit split into two smaller units, only the boundaries of the original unit would be present in the harmonized geography. IPUMS USA, IPUMS International, and IPUMS Terra offer harmonized geographies^20^. IPUMS NHGIS applies interpolation to provide estimates of 1990 and 2000 statistical data for 2010 boundaries. Interpolation involves reaggregating statistics from 2000 block level data to construct values for coarser 2010 geographic levels, including spatial allocations of population where 2000 blocks are split across 2010 units^21^.

### Aggregate time series tables

While microdata may be harmonized over time by matching corresponding codes, aggregate data can pose additional challenges in how sets of responses are grouped from one census to another. For example, age is generally trivial to harmonize in microdata, since each individual reports their age in years. In aggregate data tables, however, one census may include a column of persons age 0 to 2, while another census includes a column of persons age 0 to 5. IPUMS NHGIS has identified sets of census years and tables for which it is possible to construct consistent quantities and provides these as time series tables^10^.

### Record linkage

Linking, or identifying the same individual as they appear across multiple censuses or surveys, enables researchers to study life course. IPUMS USA provides linkages from microdata samples for the 1850–1930 censuses to complete count microdata from the 1880 census, and NAPP provides linkages between the 1865, 1875, and 1900 Norwegian censuses, with additional linkages underway. These linkages are based on matching individual characteristics—name, year and place of birth, and race—on records from each census^7,22–24^. IPUMS CPS provides unique identifiers for individuals and households based on technical information from the Current Population Survey to enable tracking of individuals throughout the eight months they participate in the survey^25^.

### Location-based integration

IPUMS Terra goes beyond harmonization within a particular type of data and incorporates integration across multiple data structures—microdata describing individuals, area-level data describing places, and grid-based raster data. By integrating across these data structures, each commonly used in certain scientific domains, IPUMS Terra facilitates cross-disciplinary research, especially dealing with human-environment interactions. IPUMS Terra leverages spatial information in each type of data structure to perform transformations across data structures, such as summarizing raster grid values that fall within a geographic unit to create area-level data, or attaching area-level data to microdata records based on the unit in which the household is located^11,26^.

### Household relationship variables

A very powerful feature of microdata is the organization of individuals into households, enabling research that addresses questions of household structure. To facilitate such research, IPUMS microdata products create relationship variables for each individual, identifying the location of the person’s mother, father, and spouse within the household and describing the individual’s co-resident children^27^. The IPUMS data extract systems leverages these variables to attach characteristics of an individual’s relatives to their record, such as spouse’s educational attainment.

## Additional information

**How to cite this article:** Kugler, T. A. & Fitch C. A. Interoperable and accessible census and survey data from IPUMS. *Sci. Data* 5:180007 doi: 10.1038/sdata.2018.7 (2018).

**Publisher’s note:** Springer Nature remains neutral with regard to jurisdictional claims in published maps and institutional affiliations.
